# Identification of allograft inflammatory factor-1 suppressing the progression and indicating good prognosis of osteosarcoma

**DOI:** 10.1186/s12891-024-07363-8

**Published:** 2024-03-23

**Authors:** Wenda Liu, Tao Shi, Di Zheng, Guangshui Ke, Jingteng Chen

**Affiliations:** 1 Department of Orthopedics, Xiangyang No.1 People’s Hospital, Hubei University of Medicine, Xiangyang, 441000 Hubei Province P. R. China; 2 Department of Orthopedics, Renmin Hospital of Wuhan University, Wuhan, 430060 Hubei Province P.R. China; 3 Department of Otolaryngology-Head and Neck Surgery, Renmin Hospital of Wuhan University, Wuhan, 430060 Hubei Province P.R. China

**Keywords:** Osteosarcoma, Allograft inflammatory factor-1, Immune invasion, Indicating good prognosis, NF-κB pathway

## Abstract

**Background:**

Osteosarcoma is one of the most common cancers worldwide. Intense efforts have been made to elucidate the pathogeny, but the mechanisms of osteosarcoma are still not well understood. We aimed to investigate the potential biomarker, allograft inflammatory factor-1 (AIF1), affecting the progression and prognosis of osteosarcoma.

**Methods:**

Three microarray datasets were downloaded from GEO datasets and one was obtained from the TCGA dataset. The differentially expressed genes (DEGs) were identified. GO and KEGG functional enrichment analyses of overlapped DEGs were performed. The PPI network of overlapped DEGs was constructed by STRING and visualized with Cytoscape. Overall survival (OS) and Metastasis free survival (MFS) were analyzed from GSE21257. Finally, the effect of the most relevant core gene affecting the progression of osteosarcoma was examined in vitro.

**Results:**

One hundred twenty six DEGs were identified, consisting of 65 upregulated and 61 downregulated genes. Only AIF1 was significantly associated with OS and MFS. It was found that AIF1 could be enriched into the NF-κB signaling pathway. GSEA and ssGSEA analyses showed that AIF1 was associated with the immune invasion of tumors. Cell experiments showed that AIF1 was underexpressed in osteosarcoma cell lines, while the malignant propriety was attenuated after overexpressing the expression of AIF1. Moreover, AIF1 also affects the expression of the NF-κB pathway.

**Conclusion:**

In conclusion, DEGs and hub genes identified in the present study help us understand the molecular mechanisms underlying the carcinogenesis and progression of osteosarcoma, and provide candidate targets for diagnosis and treatment of osteosarcoma.

**Supplementary Information:**

The online version contains supplementary material available at 10.1186/s12891-024-07363-8.

## Introduction

Osteosarcoma, derived from mesenchymal tissue, is the most common primary malignant bone tumor and usually occurs in adolescents with high malignancy and poor prognosis [1]. In the past few decades, the treatment of osteosarcoma has progressed from surgical resection to chemotherapy and limb salvage treatment. Thus the survival rate of patients has also been greatly improved. However, the latest studies found that patients with osteosarcoma have complicated chemotherapy resistance and lung metastases during treatment periods and the 5-year survival rate is less than 20% [2–4]. The treatment of osteosarcoma encounters a bottleneck and brings huge pain and economic burden to patients. Thus, the mechanisms of resistance and metastasis of osteosarcoma are supposed to be elucidated, further research is urgently needed to discover new treatment options.

The allograft inflammatory factor-1 (AIF1), located in the major histocompatibility complex (MHC) class III region on chromosome 6p21.3, is a 17 kDa calcium-binding protein produced by monocytes, macrophages, and lymphocytes, which is induced by INF-γ [5]. Studies have demonstrated that AIF1 plays a chemoattractant for macrophages and modulates inflammatory processes [6]. Dysregulated expression of AIF1 was observed in several diseases, including endometriosis, breast cancer, rheumatoid arthritis, and fibrosis [5, 7–9]. In addition, AIF1 may play a significant role in the pathophysiology and progression of gastric cancer, hepatocellular carcinoma as well as colorectal cancer [10–12]. However, it has not yet been reported whether AIF1 is also associated with the development of osteosarcoma.

In the present study, we explored a potential prognostic biomarker, AIF1, of osteosarcoma. First, we used bioinformatics analysis to obtain differentially expressed genes (DEGs) between osteosarcoma tissues and normal osteoblasts, 3 mRNA microarray datasets from Gene Expression Omnibus (GEO) were downloaded and analyzed. Subsequently, key genes involved in the molecular mechanisms underlying carcinogenesis and progression of osteosarcoma were picked out using bioinformatics methods, such as Gene Ontology (GO), Kyoto Encyclopedia of Genes and Genomes (KEGG) pathway enrichment analysis and protein-protein interaction (PPI) network analysis. Following, the prognostic value of the genes was evaluated using survival analysis of another gene expression data from GEO, and we found only AIF1 was associated with overall survival and metastases-free survival amongst these biomarkers. KEGG analysis showed that AIF1 was related to the NF-κB pathway. GSEA and ssGSEA analysis showed that AIF1 affected the immune microenvironment of osteosarcoma and was correlated with immune checkpoints. So, we further performed in vitro experiments to elaborate on the potential roles of AIF1 in proliferative and invasion ability and the influence in the NF-κB pathway of osteosarcoma cells. In conclusion, we demonstrated that AIF1 functions as a tumor suppressor, and loss of AIF1 expression may represent a novel indicator for the progression and prognosis of osteosarcoma.

## Materials and methods

### Microarray data

RNA sequencing data (RNA-seq) in fragment per kilobase method (FPKM) format, together with accompanying clinical information, was collected from The Cancer Genome Atlas (https://portal.gdc.cancer.gov/) for a total of 88 patients diagnosed with osteosarcoma. The Gene Expression Omnibus (GEO) is a publicly accessible repository for high-throughput gene expression data, including information on chips and microarrays. It can be accessed at the following URL: http://www.ncbi.nlm.nih.gov/geo. The following four gene expression datasets were downloaded: GSE33382 [13], GSE14359 [14], GSE12865 [15], and GSE21257 [16]. The dataset GSE33382 utilized the Illumina human-6 v2.0 expression bead chip platform (GPL10295) to analyze gene expression in three osteoblast tissues and 84 osteosarcoma samples. The dataset GSE14359 utilized the Affymetrix Human Genome U133A Array Platform (GPL96) to examine gene expression. It consisted of 18 osteosarcoma samples and 2 osteoblast samples. The GSE12865 dataset utilized the Affymetrix Human Gene 1.0 ST Array as its platform, encompassing 14 samples. This included 2 samples of osteoblasts and 12 samples of osteosarcoma. The GSE21257 dataset comprised 34 pre-chemotherapy biopsies obtained from osteosarcoma patients who exhibited metastatic progression throughout 5 years, alongside 19 samples from patients who did not experience metastasis. The dataset comprised clinical data about both overall survival and metastasis-free survival.

### Data processing

The GEO2R tool, available at http://www.ncbi.nlm.nih.gov/geo/geo2r, was employed to perform differential gene expression analysis to filter the differentially expressed genes (DEGs) between osteosarcoma and osteoblast samples. The GEO2R web program enables users to do comparisons of multiple datasets within a GEO series to identify differentially expressed genes (DEGs) across different experimental conditions. To reconcile the limitations posed by false positives and the need to identify pertinent genes, the adjusted *P*-value (adj.P) and Benjamini methods were employed. Genes that possessed multiple probe sets or probe sets lacking corresponding gene symbols were either averaged or excluded, as appropriate. Statistical significance was operationally defined in this study as an adjusted *p*-value less than 0.05 and an absolute logarithmic fold change greater than 1.

### KEGG and GO enrichment analyses of DEGs

The Database for Annotation, Visualization, and Integrated Discovery (DAVID; http://david.nicifcrf.gov) (version 6.8) offers a comprehensive assortment of functional annotation tools. This web-based program aids researchers in interpreting the biological relevance of numerous genes and proteins [17, 18]. The KEGG database resource facilitates the understanding of biological systems and their high-level functions through the utilization of extensive molecular information generated by high-throughput experimental techniques [19]. Ontology is a prevalent bioinformatics approach employed to provide comprehensive insights into the functional attributes of genetic products, with Gene Ontology (GO) being a prominent example [20]. The role of differentially expressed genes (DEGs) was examined through biological investigations using the DAVID online database. The cut-off value was considered to be *P* < 0.05.

### PPI network construction and module analysis

The Search Tool for the Retrieval of Interacting Genes (STRING; http://string-db.org) (version 11.0) online database was utilized to predict the protein-protein interaction (PPI) network [21]. The investigation of the functional associations between proteins may yield valuable insights into the mechanisms behind the onset or advancement of diseases [22]. The construction of the protein-protein interaction (PPI) network for the present study was facilitated by utilizing the STRING database. An interaction was considered to be statistically significant if its combined score exceeded 0.4. Cytoscape, an open-source software platform (version 3.7.2), can be employed for the visualization of intricate networks and their integration with diverse attribute data [23]. The Molecular Complex Detection (MCODE) application, namely version 1.5 or later, is a tool that may be utilized in conjunction with Cytoscape software. Its primary function is to identify densely connected regions within a given network by employing clustering algorithms based on the network’s topology [24]. The construction of protein-protein interaction (PPI) networks was facilitated by employing Cytoscape, while the identification of the most significant module within these networks was accomplished through the utilization of MCODE. The selection criteria included MCODE scores greater than 2, a degree cut-off of 2, a node score cut-off of 0.2, a maximum depth of 100, and a k-score of 2. The DAVID tool was subsequently employed to conduct KEGG and GO analyses on the genes encompassed within this module.

### Hub genes selection and analysis

The selection of hub genes was made with a minimum threshold of 10. The investigation involved the analysis of a network of genes and their co-expression genes using the web platform cBioportal (http://cbioportal.org), which serves as a hub for this purpose [25, 26]. The selection of hub genes was performed with a minimum threshold of 10. The cBioportal web platform (http://cbioportal.org) was utilized to investigate a network consisting of genes and their co-expression genes.

### Kaplan-Meier survival analysis

The predictive relevance of the genes in the present study was validated using GraphPad Prism 5. This validation was conducted by Kaplan-Meier survival analysis, utilizing clinical data obtained from the GSE21257 dataset. The patients were categorized into two groups for statistical analysis based on their gene expression levels. The patients were stratified into two cohorts based on their respective levels of expression: individuals with values surpassing the median, and those with levels below it. The assessment of the predictive value of the genes was conducted utilizing the log-rank test, which is also referred to as the Mantel-Cox test.

### Screening of differential genes between high and low expression groups of AIF1

The AIF1 expression levels of the TCGA cohort were divided into two categories based on high and low expression. By using the criteria of an absolute log2 fold change greater than 0.5 and a *p*-value less than 0.05, we successfully discerned the differentially expressed genes (DEGs) that exhibited distinct expression patterns between the two groups. Subsequently, a heatmap was generated to visually represent the expression patterns of the differentially expressed genes (DEGs) among the various samples. The hub genes were identified by simulating the protein-protein interaction (PPI) network of the differentially expressed genes between the two groups using the STRING web-based database (string-interaction.org).

### GSEA

To provide a comprehensive understanding of the mechanisms underlying differential gene expression, we employed Gene Set Enrichment Analysis (GSEA) on the high- and low-expression cohorts. The objective of this study was to ascertain the potential involvement of these genes in certain biological pathways or activities. The GSEA algorithm was utilized to analyze the gene set database “c2.cp.kegg.v7.0.symbols.gmt”.

### Analysis of immune function between high- and low-expression groups

The immunological scores, estimate scores, stromal scores, and tumor purity of osteosarcoma patients were quantified in two distinct expression groups to investigate the impact of AIF1 on the tumor microenvironment (TME). The expression data of each sample was analyzed using the CIBERSORT approach to determine the relative abundance of 22 distinct immune cell types. The differences in the quantity of immune cells between the two groups of gene expression were subsequently visualized using R software tools. Additionally, an examination was conducted on the correlation between the infiltration of immune cells and the osteosarcoma samples. Furthermore, the TCGA dataset was utilized for conducting a single-sample gene set enrichment analysis (ssGSEA). This study elucidated the variations in immune function ratings and the expression of the 22 invading cell types between the high- and low-expression groups. Finally, we examined the correlation between the expression of immunological checkpoints in osteosarcoma and the expression of AIF1.

### Cell culture and transfection

The 143B, MG63, HOS, and hFOB1.19 cell lines were donated by the Shanghai Institute of Biochemistry and Cell Biology, Chinese Academy of Sciences (Shanghai, China) to conduct the in vitro assays. The cells were cultured in Dulbecco’s modified Eagle’s medium (Gibco-BRL Life Technologies, Grand Island, NY, USA), supplemented with 10% fetal bovine serum (Tianhang Biological Technology, Hangzhou, China), 100 U/ml penicillin, and 100 μg/ml streptomycin. Each cell was maintained at a temperature of 37 °C within a controlled atmosphere containing 5% carbon dioxide (CO2). Lentiviruses obtained from OBiO (Shanghai, China) were employed to generate lentivirus transfection constructs to induce AIF1 overexpression (LV-AIF1) and serving as an empty control (LV-Control).

### Cell growth assay

Cell proliferation was determined by measuring the number of viable cells at various time points following transfection. This was accomplished using a colorimetric water-soluble tetrazolium salt assay, specifically the Cell Counting Kit-8 from Dojingdo Laboratories in Kumamoto, Japan.

### Scratch migration assay

LV-AIF1 cells, an empty control, and a control were transfected in a 6-well plate. The cells were collected 48 hours post-transfection by gently scraping them using the tiny tip of a 10-μl pipette, marking the starting time as 0. Following many washes of the plates with phosphate-buffered saline (PBS) to remove any cells that were not adherent, the entire growth medium was subsequently introduced and subjected to incubation. Cell migration into the damaged region was observed and recorded on camera after 36 hours.

### Transwell invasion assay

The invasion tests were conducted using Matrigel (Corning, USA) and transwell chambers (Corning, USA). A volume of 500 μl (μL) of medium containing 20% fetal bovine serum (FBS) was added to the lower chamber. An 80 μL mixture was prepared by combining Matrigel and a medium in a ratio of 1:8, which was subsequently added to the top chamber. After the solidification of the mixture, the upper chamber was filled with 200 μL of medium containing 1 × 105 cells. The upper chamber was treated with a 4% paraformaldehyde solution and stained using a 1% crystal violet solution following a 48-hour incubation period at a temperature of 37 °C with a 5% concentration of CO2. The invading cells in the top chamber were measured and analyzed using an Olympus inverted microscope manufactured in Japan. The analysis was conducted using the ImageJ program.

### Western blotting

Total proteins were extracted from osteosarcoma cells and tissues. The protein concentration was measured using a BCA kit (Servicebio Technology, Wuhan, China). Following separation on a 10% or 12% sodium dodecyl sulfate-polyacrylamide gel electrophoresis (SDS-PAGE), approximately 10–25 μg of total protein were subsequently deposited onto a polyvinylidene fluoride membrane. After the blocking step, the membrane was subjected to be incubated with primary antibodies overnight at 4 °C, while concurrently being washed with TBST (tris-buffered saline with tween). Subsequently, the membrane was subjected to a rinsing procedure using TBST and subsequently left at ambient temperature for 1 hour, during which the secondary antibodies were incubated. Subsequently, the bands were seen using an ECL kit manufactured by Thermo Fisher Scientific. The primary antibodies utilized in this study consisted of a monoclonal mouse anti-tubulin antibody (diluted at 1:3000, obtained from Servicebio, Wuhan, China) and a polyclonal rabbit anti-AIF1 antibody (diluted at 1:1000, obtained from Bioss, Beijing, China).

### Statistical analysis

The Fisher’s exact test was employed to evaluate the association between AIF1 expression and clinicopathological indicators. The study adopted the Kaplan-Meier technique to assess the probability of variations in osteosarcoma over time. Additionally, a log-rank test was utilized to estimate the statistical significance of these variations. The statistical analyses were conducted using SPSS 16.0 software (SPSS, Inc., Chicago, IL, USA). A statistically significant difference was operationally defined as a difference with a *P*-value below the threshold of 0.05.

## Results

### Identification of DEGs in osteosarcoma

**Fig. 1:**
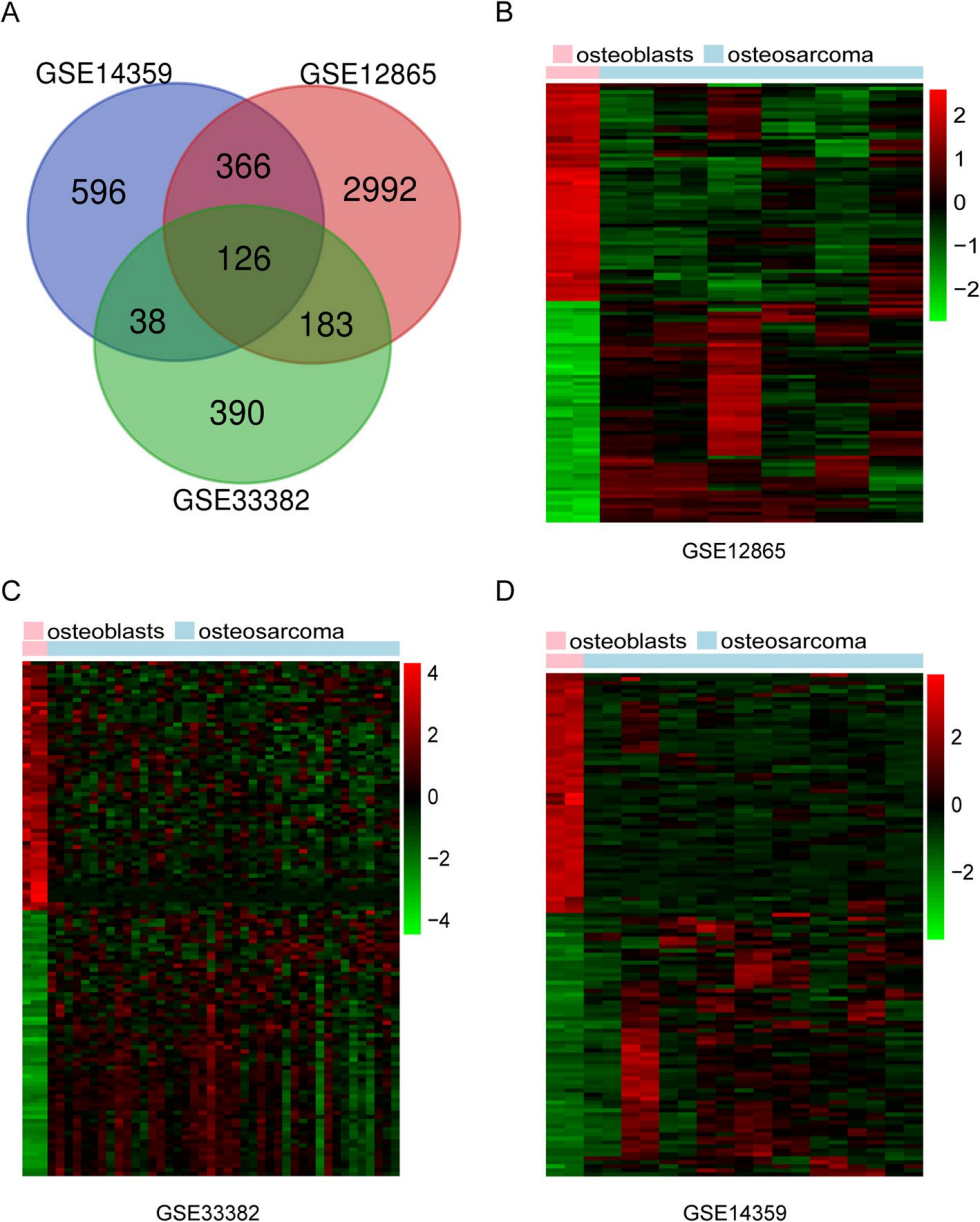
Venn diagram and heatmap images. **A** DEGs were selected with a |log_2_FC|>1 and *adj.P*<0.05 among the mRNA expression profiling sets GSE33382, GSE14359, and GSE12865. The 3 datasets showed an overlap of 126 genes. **B**-**D** The heatmap images of DEGs of osteoblasts vs. osteosarcoma from 3 mRNA expression profiling sets GSE33382, GSE14359, and GSE12865, respectively. Upregulated genes were marked in red; downregulated genes were marked in green

After standardization (with |log_2_FC|>1 and *adj.P*<0.05 as the cut-off point) of the microarray results, DEGs(737 in GSE33382，1126 in GSE14359 and 3667 in GSE12865) were identified (Table 1). The overlap among the 3 datasets contained 126 genes as shown in the Venn diagram (Fig. 1A), consisting of 65 upregulated genes and 61 downregulated genes between osteosarcoma tissues and normal osteoblasts (Fig. 1B-D).

**Table 1:** Summary of osteosarcoma microarray datasets from different gene expression omnibus dataset

	Series	Platform	Affymetrix gene chip	Sample	Normal	Osteosarcoma
1	GSE33382	GPL10295	Illumina human-6 v2.0 expression bead chip	87	3	84
2	GSE14359	GPL96	Affymetrix Human Genome U133A Array	20	2	18
3	GSE12865	GPL6244	Affymetrix Human Gene 1.0 ST Array	14	2	12

### KEGG and GO enrichment analysis of DEGs

**Fig. 2:**
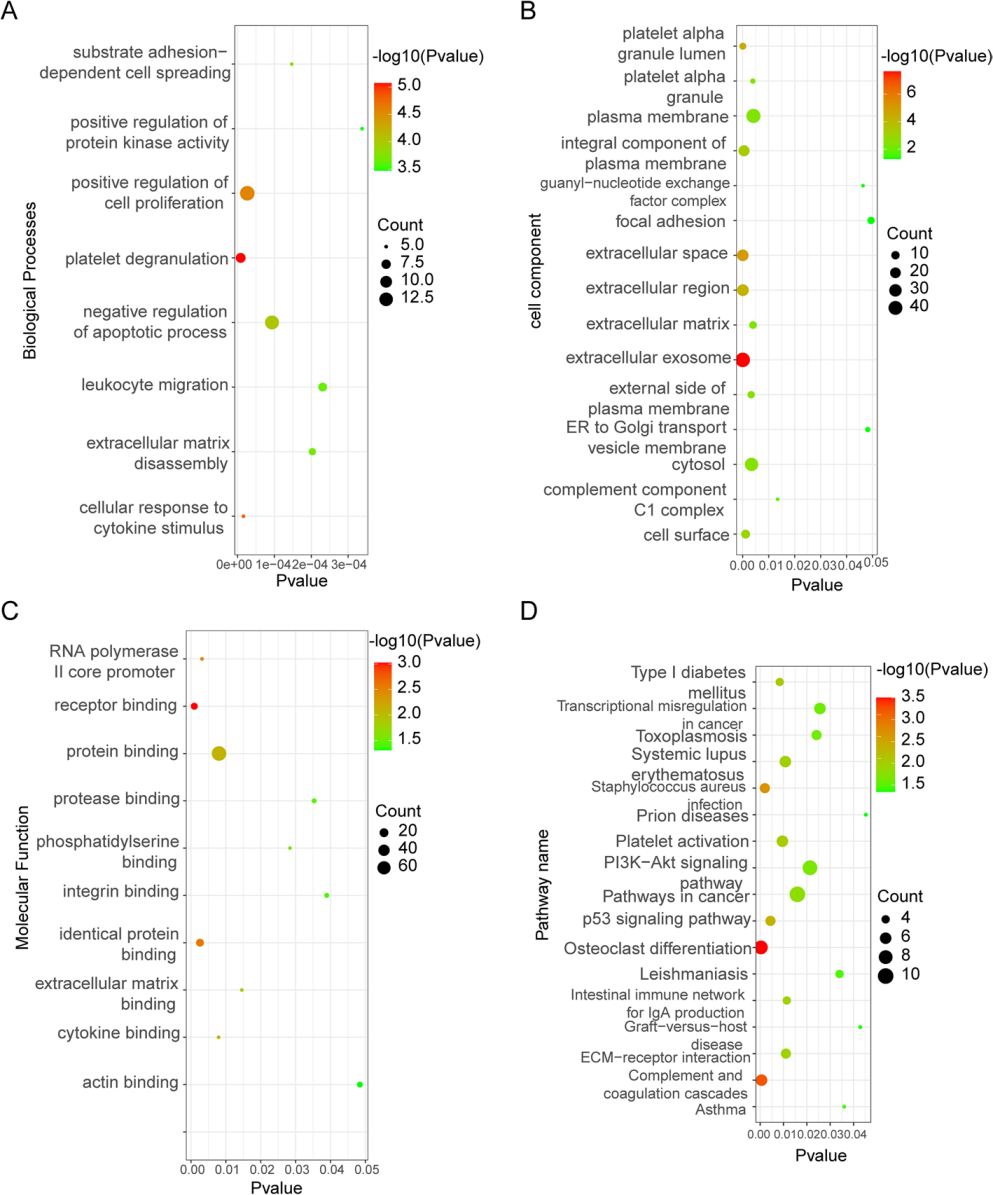
GO and KEGG enrichment analysis of DEGs. **A** Biological process, **B** Cellular components, **C** Molecular functions, **D** KEGG pathway

To analyze the biological classification of DEGs, functional and pathway enrichment analyses were performed using DAVID. GO functional and KEGG pathways of which *P*<0.05 were selected as significant results (Tables 2 and 3).GO analysis results showed that changes in biological processes (BP) of DEGs were significantly enriched in platelet degranulation, positive regulation of cell proliferation, and negative regulation of apoptotic process (Fig. 2A). Changes in molecular function (MF) were mainly enriched in receptor binding， identical protein binding, and protein binding (Fig. 2B). Changes in cell component (CC) of DEGs were mainly enriched in extracellular exosome extracellular space， extracellular region， an integral component of the plasma membrane， cytosol, and plasma membrane (Fig. 2C). KEGG pathway analysis revealed that the DEGs were mainly enriched in Osteoclast differentiation, Complement and coagulation cascades, and Staphylococcus (Fig. 2D) with detailed information in Table 3.

**Table 2:** GO enrichment analysis for the differentially expressed mRNAs

Category	Term	Description	Count	*P*-value
GO BP	GO:0002576	platelet degranulation	8	8.49E-06
GO BP	GO:0071345	cellular response to cytokine stimulus	5	1.59E-05
GO BP	GO:0008284	positive regulation of cell proliferation	14	2.64E-05
GO BP	GO:0043066	negative regulation of the apoptotic process	13	9.35E-05
GO BP	GO:0034446	substrate adhesion-dependent cell spreading	5	1.47E-04
GO BP	GO:0022617	extracellular matrix disassembly	6	2.03E-04
GO BP	GO:0050900	leukocyte migration	7	2.31E-04
GO BP	GO:0045860	positive regulation of protein kinase activity	5	3.38E-04
GO CC	GO:0070062	extracellular exosome	45	2.24E-08
GO CC	GO:0005615	extracellular space	25	9.84E-06
GO CC	GO:0031093	platelet alpha granule lumen	6	3.43E-05
GO CC	GO:0005576	extracellular region	26	6.31E-05
GO CC	GO:0005887	an integral component of the plasma membrane	22	4.90E-04
GO CC	GO:0009986	cell surface	12	1.11E-03
GO CC	GO:0009897	external side of the plasma membrane	7	3.25E-03
GO CC	GO:0005829	cytosol	36	3.40E-03
GO CC	GO:0031091	platelet alpha granule	3	3.90E-03
GO CC	GO:0031012	extracellular matrix	8	3.97E-03
GO CC	GO:0005886	plasma membrane	42	4.13E-03
GO CC	GO:0005602	complement component C1 complex	2	1.35E-02
GO CC	GO:0032045	guanyl-nucleotide exchange factor complex	2	4.63E-02
GO CC	GO:0012507	ER to Golgi transport vesicle membrane	3	4.81E-02
GO CC	GO:0005925	focal adhesion	7	4.94E-02
GO MF	GO:0005102	receptor binding	10	9.42E-04
GO MF	GO:0042802	identical protein binding	14	2.57E-03
GO MF	GO:0000983	RNA polymerase II core promoter sequence-specific	3	3.16E-03
GO MF	GO:0019955	cytokine binding	3	7.92E-03
GO MF	GO:0005515	protein binding	77	8.04E-03
GO MF	GO:0050840	extracellular matrix binding	3	1.46E-02
GO MF	GO:0001786	phosphatidylserine binding	3	2.84E-02
GO MF	GO:0002020	protease binding	4	3.53E-02
GO MF	GO:0005178	integrin binding	4	3.89E-02
GO MF	GO:0003779	actin binding	6	4.84E-02

**Table 3:** KEGG pathway enrichment analysis for the differentially expressed mRNAs

Category	Term	Description	Count	*P*-value
pathway	hsa04380	Osteoclast differentiation	8	3.13E-04
pathway	hsa04610	Complement and coagulation cascades	6	6.00E-04
pathway	hsa05150	*Staphylococcus aureus* infection	5	2.01E-03
pathway	hsa04115	p53 signaling pathway	5	4.42E-03
pathway	hsa04940	Type I diabetes mellitus	4	8.38E-03
pathway	hsa04611	Platelet activation	6	9.55E-03
pathway	hsa05322	Systemic lupus erythematosus	6	1.08E-02
pathway	hsa04512	ECM-receptor interaction	5	1.11E-02
pathway	hsa04672	Intestinal immune network for IgA production	4	1.14E-02
pathway	hsa05200	Pathways in cancer	10	1.60E-02
pathway	hsa04151	PI3K-Akt signaling pathway	9	2.14E-02
pathway	hsa05145	Toxoplasmosis	5	2.42E-02
pathway	hsa05202	Transcriptional misregulation in cancer	6	2.56E-02
pathway	hsa05140	Leishmaniasis	4	3.40E-02
pathway	hsa05310	Asthma	3	3.60E-02
pathway	hsa05332	Graft-versus-host disease	3	4.29E-02
pathway	hsa05020	Prion diseases	3	4.53E-02

### PPI network construction and module analysis

**Fig. 3:**
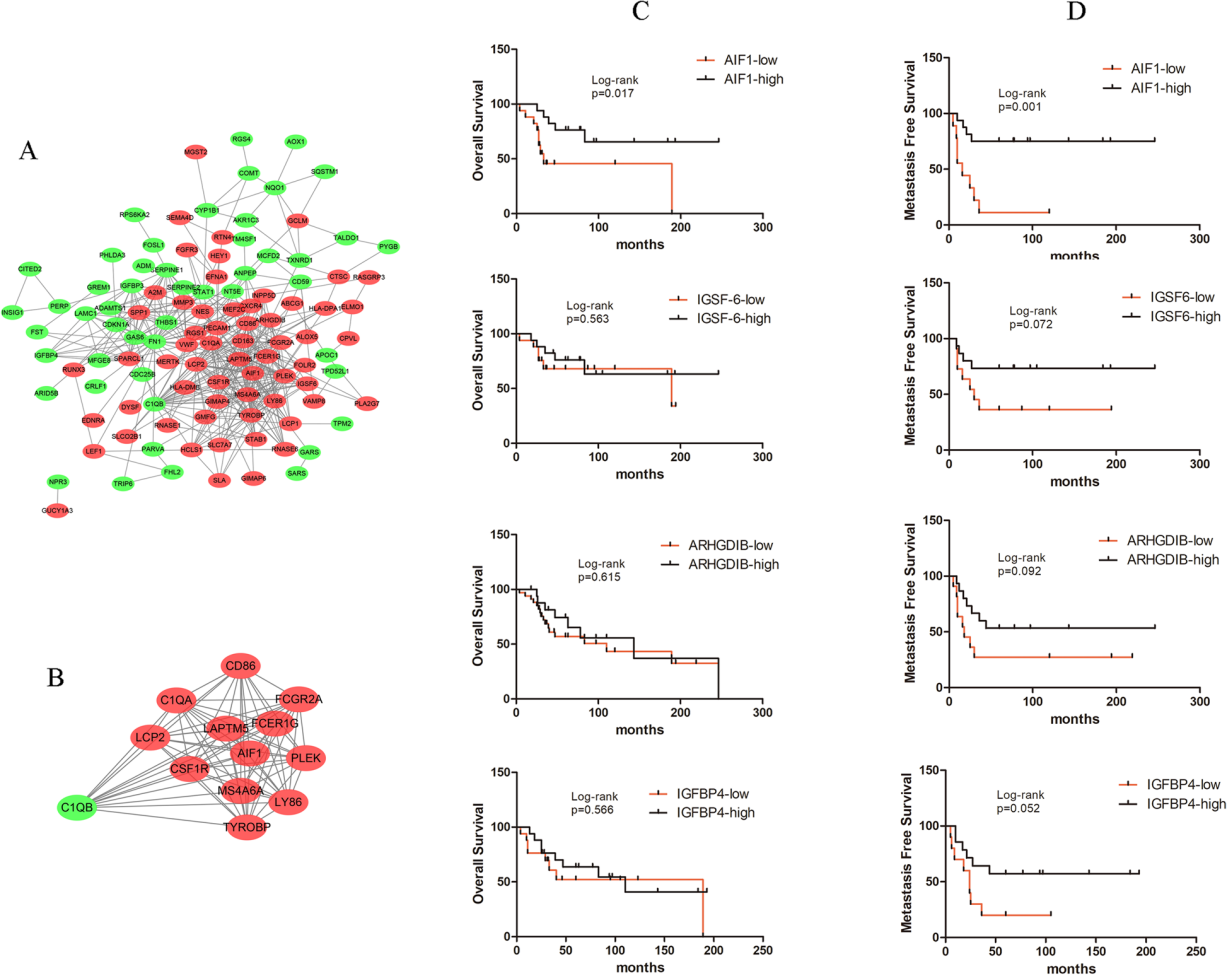
PPI network, the most significant module of DEGs and clinical association of hub genes. **A** The PPI network of DEGs was selected using Cytoscape. **B** The most significant module was obtained from the PPI network with 13 nodes and 76 edges. Upregulated genes were marked in red; downregulated genes were marked in green. **C** Overall survival curves for genes associated with the hub genes (**D**) Metastasis-free survival for genes associated with the hub genes

The PPI network of DEGs was constructed (Fig. 3A) and the most significant module was obtained using a Cystoscope (Fig. 3B). Among them, we have identified that AIF1, ARHGDIB and IGSF6 were up-regulated in DEG analysis, however, in which IGFBP4 was down-regulated. The functional analyses of genes involved in this module were analyzed using DAVID. Results showed that genes in this module were mainly enriched in Osteoclast differentiation, integrin-mediated signaling pathway, and *Staphylococcus aureus* infection (Table 4).

**Table 4:** GO and KEGG pathway enrichment analysis of DEGs in the most significant module

Pathway ID	Pathway Description	Count	*P*-value
GO:0007229	integrin-mediated signaling pathway	3	9.56E-04
GO:0002283	neutrophil activation is involved in the immune response	2	2.89E-03
GO:0045576	mast cell activation	2	3.61E-03
GO:0046488	phosphatidylinositol metabolic process	2	5.77E-03
GO:0031529	ruffle organization	2	7.93E-03
GO:0006911	phagocytosis, engulfment	2	8.65E-03
GO:0045087	innate immune response	3	1.11E-02
GO:0006958	complement activation, classical pathway	2	1.15E-02
GO:0042102	positive regulation of T cell proliferation	2	2.86E-02
GO:0032587	ruffle membrane	2	3.06E-02
GO:0005581	collagen trimer	2	3.63E-02
GO:0007169	transmembrane receptor protein tyrosine kinase signaling pathway	2	4.54E-02
bta04380	Osteoclast differentiation	4	2.87E-04
bta05150	*Staphylococcus aureus* infection	3	1.63E-03
bta04650	Natural killer cell-mediated cytotoxicity	3	6.27E-03
bta05322	Systemic lupus erythematosus	3	1.41E-02
bta05020	Prion diseases	2	3.34E-02

### Hub gene selection and analysis

A total of 4 genes were identified as hub genes with degrees ≥10. The names, abbreviations, and functions for these hub genes are shown in Table 5. Subsequently, the overall survival and metastasis-free survival analysis of the hub genes obtained from GSE21257 datasets were performed using the Kaplan-Meier curve. As shown in Fig. 3C and D, osteosarcoma patients with high expression of AIF1 alteration showed better overall survival and metastasis-free survival; while the other hub genes showed no significance with either overall survival or metastasis-free survival of osteosarcoma patients. The univariate Cox regression analysis showed that AIF1 is an independent risk factor (Supplementary Fig. S1). Taking the phenomenon above, we suspected that activation of AIF1 is associated with a better prognosis in osteosarcoma patients.

**Table 5:** Functional roles of 5 hub genes with degree ≥10

No.	Gene symbol	Full name	Function
1	AIF1	Allograft inflammatory factor 1	Actin-binding protein that enhances membrane ruffling and RAC activation. Plays a role in RAC signaling and phagocytosis.
2	IGFBP4	Insulin-like growth factor binding protein 4	IGF-binding proteins prolong the half-life of the IGFs and have been shown to either inhibit or stimulate the growth-promoting effects of the IGFs on cell culture.
3	ARHGDIB	Rho GDP dissociation inhibitor beta	Regulates the GDP/GTP exchange reaction of the Rho proteins; regulates reorganization of the actin cytoskeleton mediated by Rho family members
4	IGSF6	Immunoglobulin superfamily member 6	It is coded entirely within the intron of METTL9 which is transcribed in the opposite strand of the DNA.

### DEGs and PPI network analyses in two expression groups

**Fig. 4:**
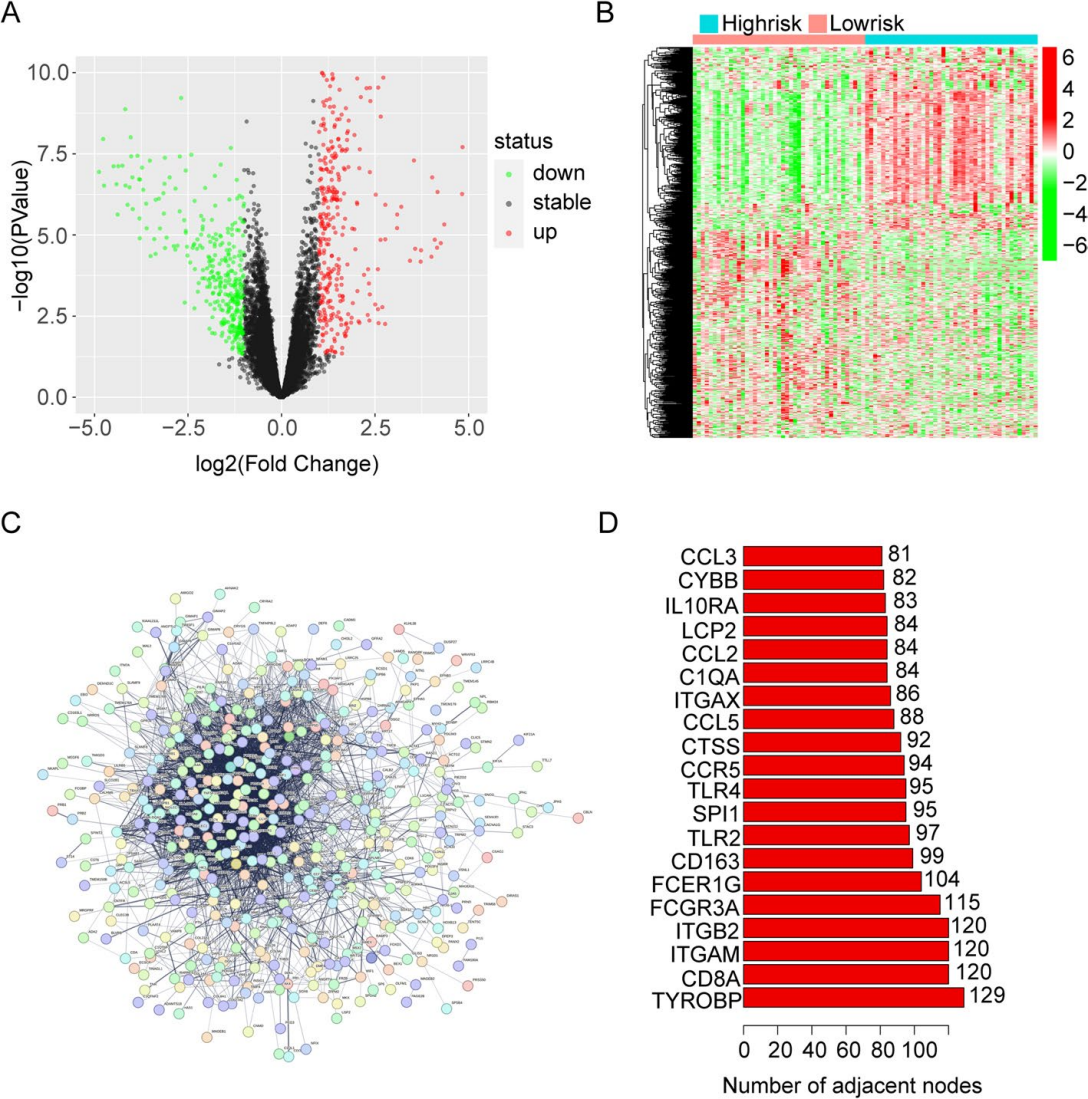
Gene differential analysis. **A** The volcano plot. **B** A heatmap was conducted. **C** PPI network. **D** The PPI network’s hub genes

In the TCGA cohort, we performed an analysis of differentially expressed genes between the high- and low-expression groups. A volcano map (Fig. 4A) was generated, revealing 748 differential genes. Among these, 397 genes were upregulated and 351 genes were downregulated in the high-expression group. A heatmap depicting the expression of these differential genes in each sample was created for subsequent analysis (Fig. 4B). Using the STRING online database and hub gene analysis, we investigated the expression patterns of the differential genes between the two expression groups (Fig. 4C-D). The result identified CD8A, VEGFA, CCR5, and FCGR3A as hub genes, indicating their significant interaction correlation.

### GO, KEGG, and GSEA analyses

**Fig. 5:**
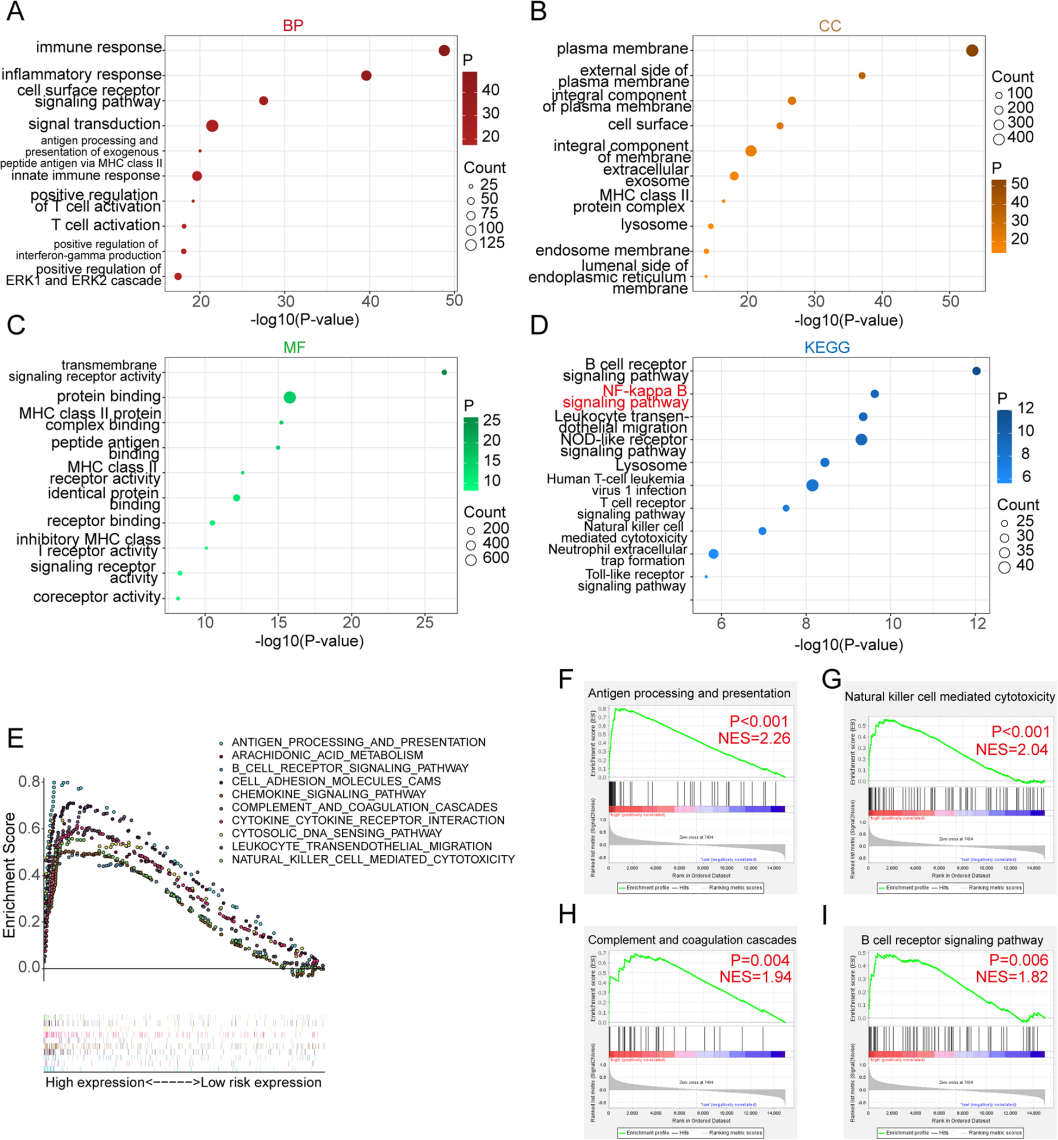
Functional enrichment and GSEA analysis. **A**-**D** GO and KEGG enrichment analysis. **E** GSEA analysis. **F** Antigen processing and presentation. **G** Natural killer cell mediated cytotoxicity. **H** Complement and coagulation cascades. **I** B cell receptor signaling pathway

Functional analyses were conducted on the differential genes, encompassing GO and KEGG analysis. The results of the GO enrichment analysis were presented in Fig. 5A-C, indicating significant findings. Biological process (BP) analysis highlighted immune response. Cellular component (CC) analysis revealed the external side of the plasma membrane. Molecular function (MF) analysis emphasized protein binding and transmembrane signaling receptor activity. Additionally, KEGG analyzed enrichment in the B cell receptor signaling pathway, NF-kappa B signaling pathway, and T cell receptor signaling pathway (Fig. 5D). These functional enrichment results further confirm the key role of AIF1 in the immunity of osteosarcoma. Then a GSEA analysis was performed, and the top 10 functions and signaling pathways were presented (Fig. 5E). These pathways included various immune-related signaling pathways that exhibited differential expression between the high- and low-expression groups. Notably, antigen processing and presentation (Fig. 5F), natural killer cell mediated cytotoxicity (Fig. 5G), complement and coagulation cascades (Fig. 5H), and B cell receptor signaling pathway (Fig. 5I) were upregulated in the low-risk group. These enrichment results suggest that AIF1 can regulate the immune microenvironment of osteosarcoma patients, and thus play an important role in the development of osteosarcoma.

### Differences in immune microenvironment

**Fig. 6:**
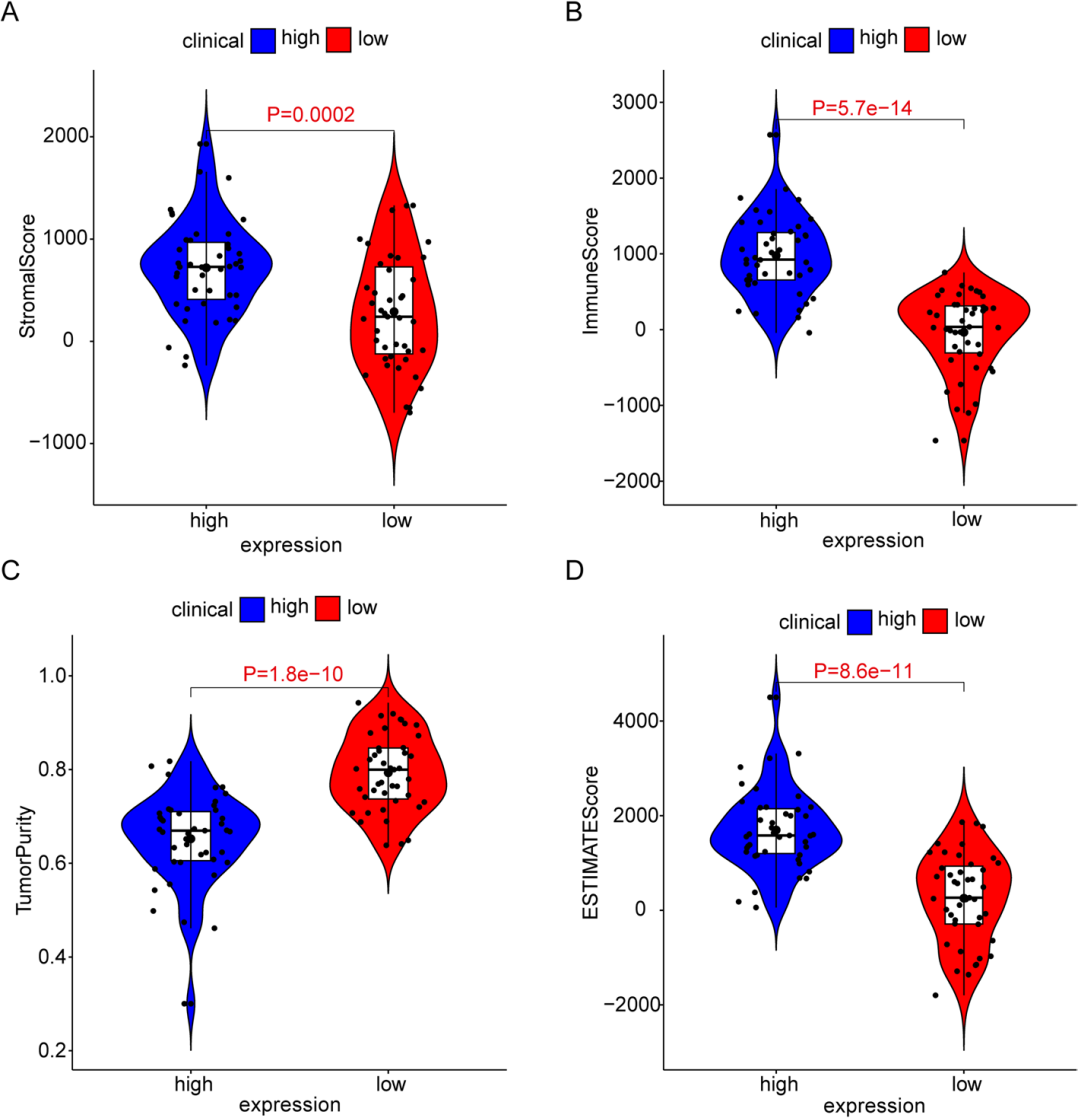
Analysis of immune-related scores. **A** The Stromal Score. **B** The Immune Score. **C** The Tumor Purity. **D** The estimated score

To explore the impact of the AIF1 on the immune microenvironment, we employed the estimation algorithm to calculate the immunological scores, estimate scores, stromal scores, and tumor purity for each sample in the TCGA cohort. The findings indicated that low-expression AIF1 exhibited significantly lower immunological scores, estimate scores, stromal scores, and higher tumor purity (Fig. 6A-D).

### Differences in immune infiltration and ssGSEA

**Fig. 7:**
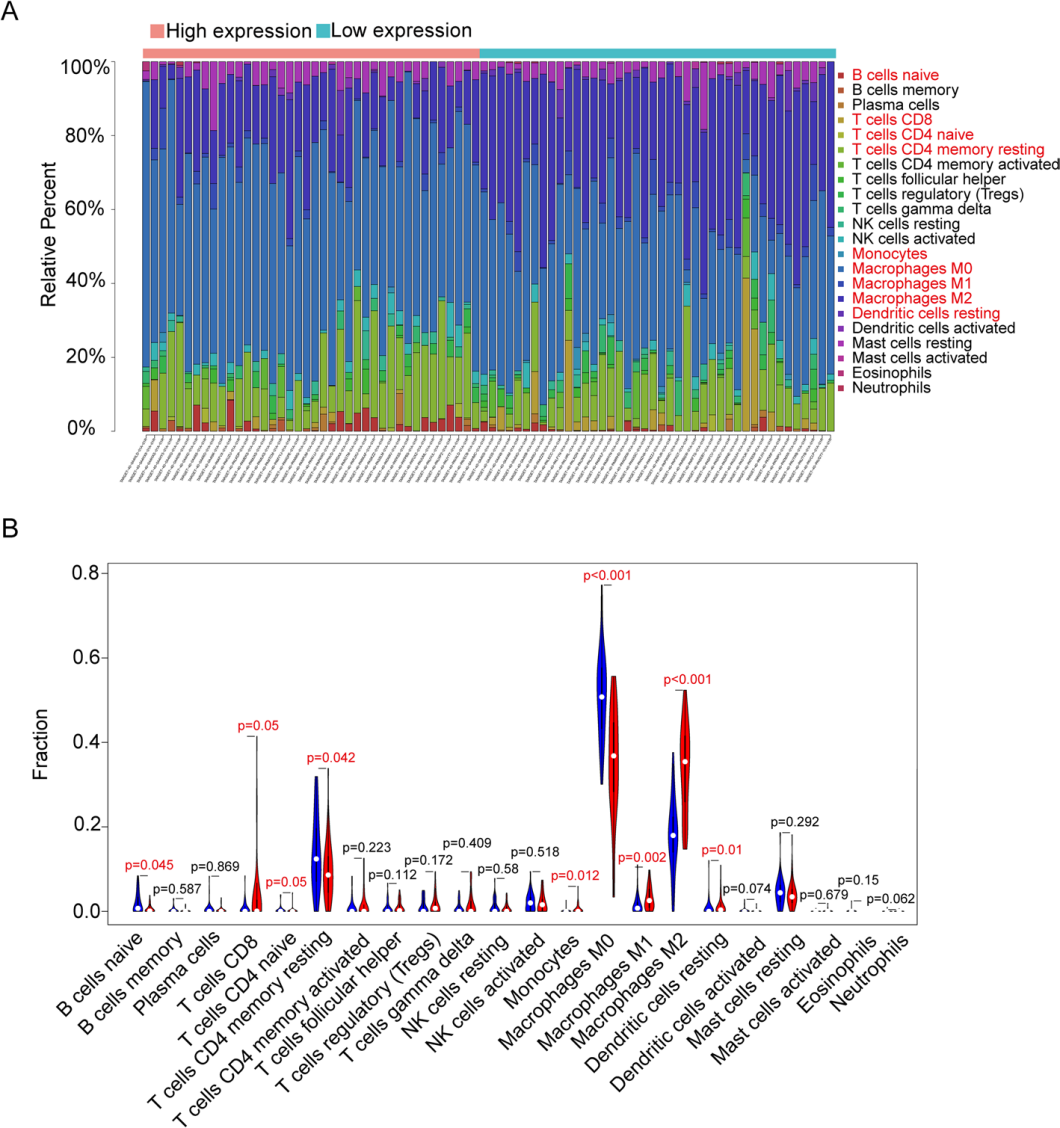
Comparison of immune cell infiltration. **A** The relative quantity of immunocyte infiltration. **B** The proportion of 22 immune cell types

**Fig. 8:**
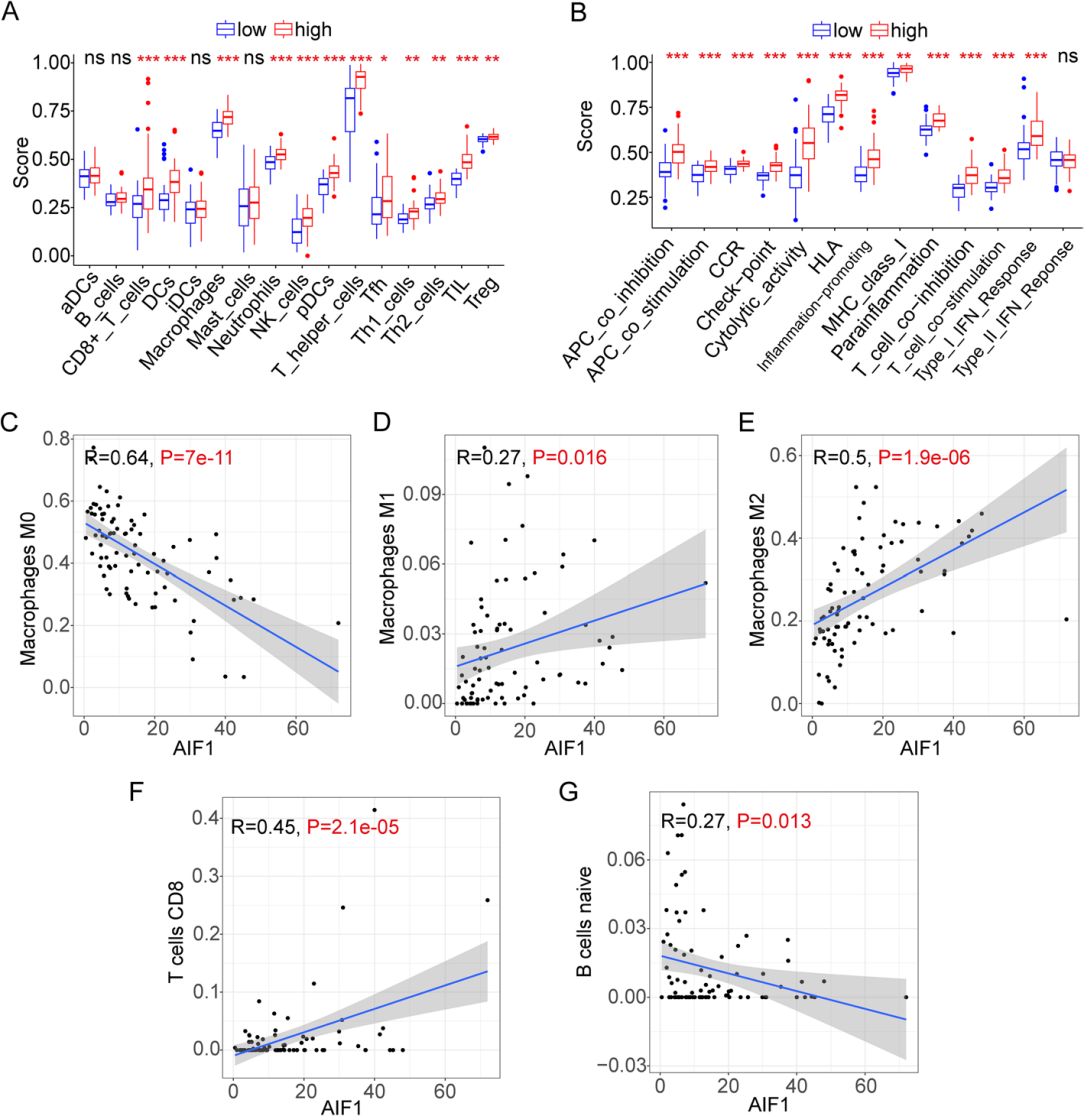
Comparison of immune cell infiltration and immune function. **A** Box plots exhibiting enrichment scores of immunocyte. **B** Box plots exhibiting enrichment scores of the related immune function. (C-G) Correlation analysis of AIF1 and immune cells. **C** Macrophages M0. **D** Macrophages M1. **E** Macrophages M2. **F** T cells CD8. **G** B cells naive

To investigate the relationship between AIF1 and immune infiltration in osteosarcoma, CIBERSORT was utilized to calculate the proportion of immune cell infiltration. A bar graph was generated to compare the immune cell proportions between the two expression groups (Fig. 7A). The analysis revealed that Macrophages M2, Macrophages M0, and Macrophages M1 were the most abundant immune cells in the osteosarcoma immune microenvironment. Additionally, an immune infiltration analysis was conducted to compare the expression differences of immune cells between the two expression groups (Fig. 7B). The study results indicated distinct proportions of immune cells in the immunological microenvironment of the osteosarcoma patients from the two expression groups. Using ssGSEA, we examined differences in immune function scores and immune cell enrichment scores between the two groups. Notably, the high-expression group exhibited higher proportions of Th2 cells and TIL cells (Fig. 8A). Furthermore, the high-expression group displayed elevated immune cell concentrations, particularly in DCs, Th1 cells, and Neutrophils. Additionally, higher CCR and checkpoint scores were observed in the high-expression group (Fig. 8B). AIF1 was positively correlated with Macrophages M1, Macrophages M2, and T cells CD8 expression and negatively correlated with Macrophages M0 and B cells naive expression (Fig. 8C-G). In summary, AIF1 can affect the immune cells and immune microenvironment of osteosarcoma patients, and then affect the occurrence and development of osteosarcoma and the prognosis of osteosarcoma patients. Therefore, the study of AIF1 may bring progress in the treatment of osteosarcoma patients.

### Activation of established cancer immune checkpoint inhibitors is positively correlated with high AIF1 expression

**Fig. 9:**
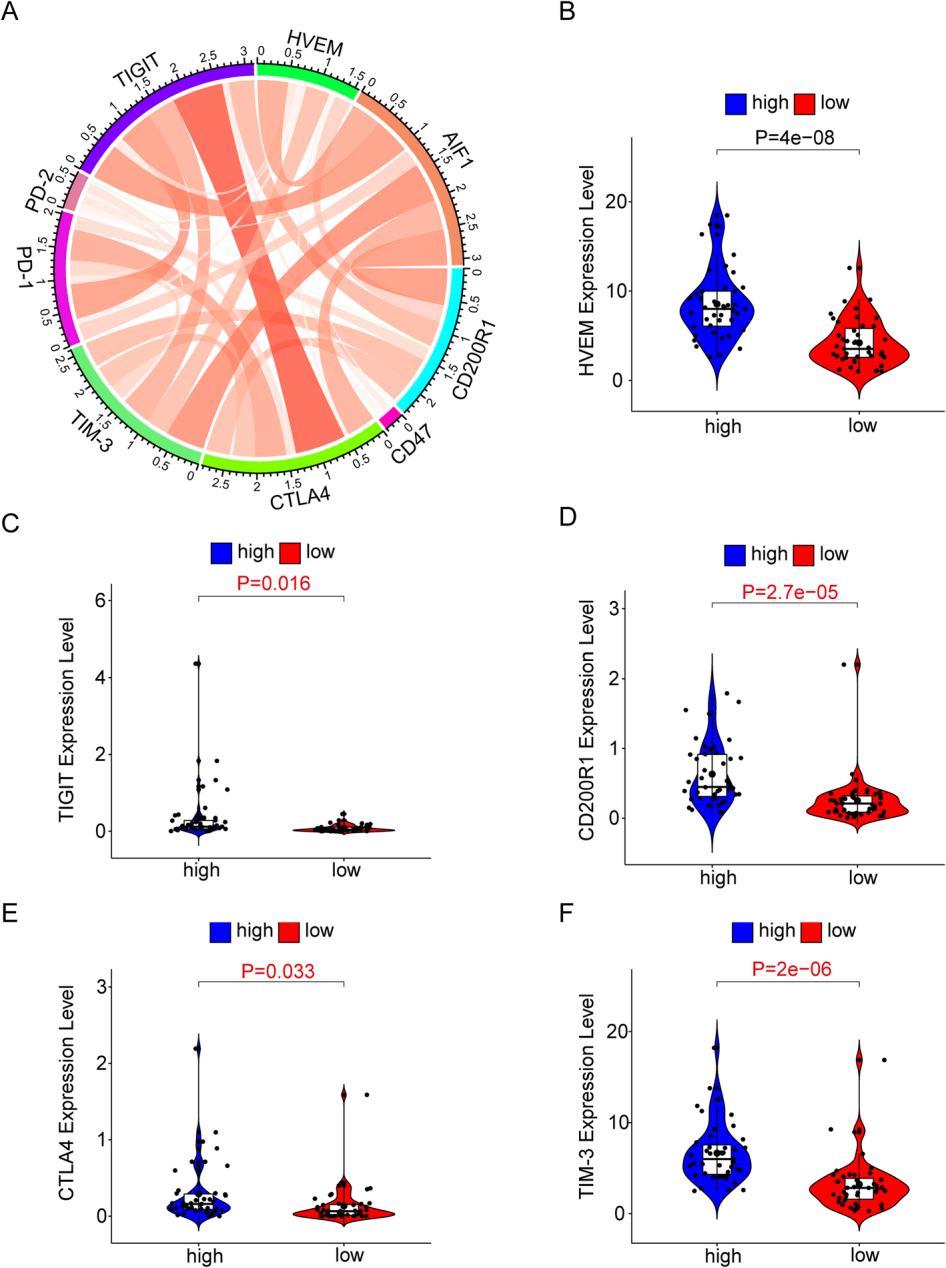
The correlation between AIF1 expression and checkpoint inhibitors in the TCGA database. **A** Pearson correlation between AIF1 and inhibitory immune checkpoints. **B** HVEM. **C** TIGIT. **D** CD200R1. **E** CTLA4. **F** TIM-3

To explore the association between AIF1 and inhibitory immune checkpoints, we examined their relationship in the TCGA databases (Fig. 9A). AIF1 demonstrated a significant positive correlation with inhibitory immune checkpoints, such as HVEM, TIM-3, TIGIT, CD200R1, and CTLA4 (Fig. 9B-F). This correlation indicates the potential involvement of AIF1 in tumor immunosuppression, leading to the inhibition of immune responses against gliomas.

### AIF1 overexpression inhibits osteosarcoma progression in vitro

**Fig. 10:**
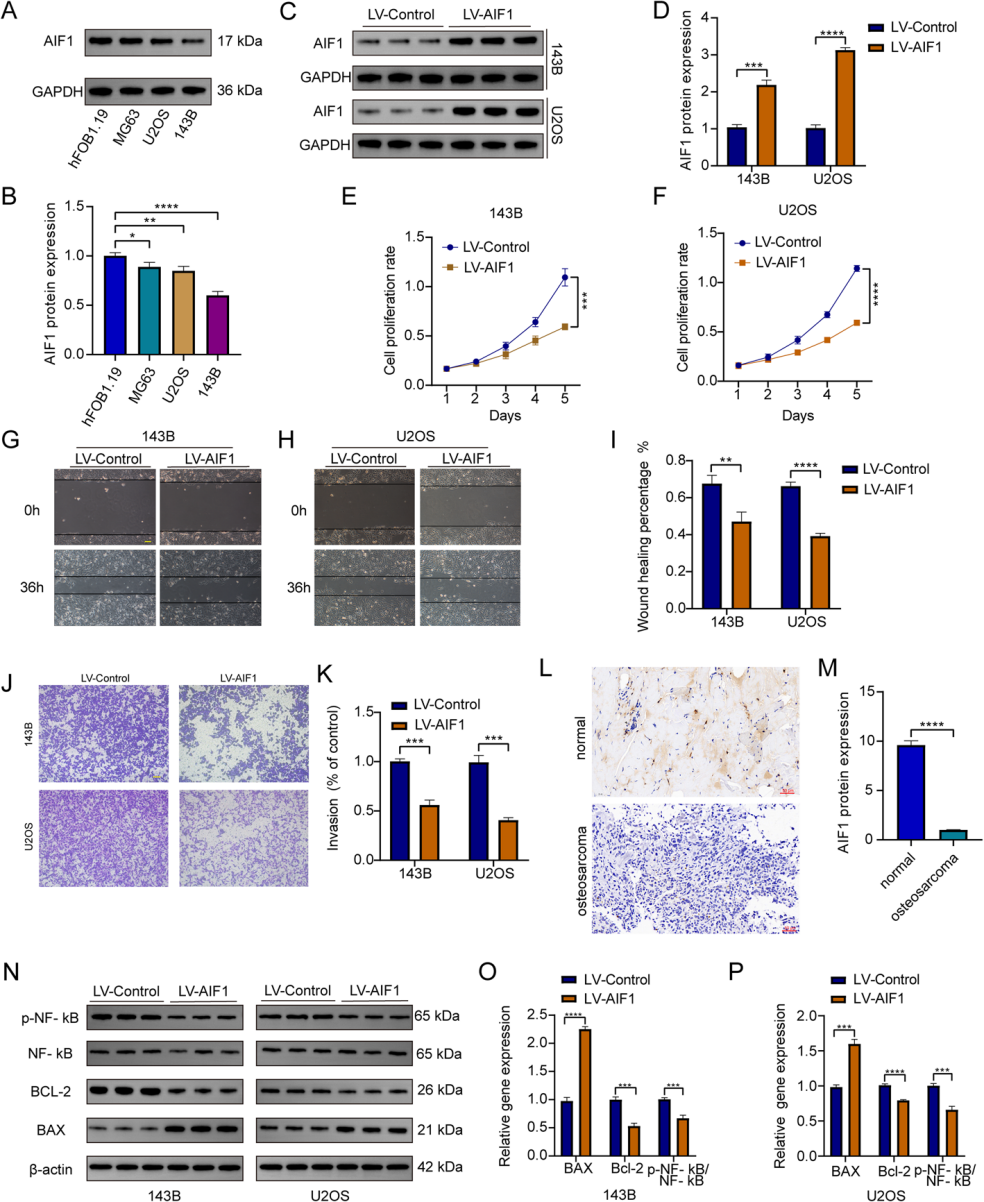
AIF1 suppressed osteosarcoma progression in vitro. **A**-**B** Western blot result showing down-regulated AIF1 expression in 143B, MG63, U2OS cell lines compared with hFOB1.19 (osteoblasts cells). **C**-**D** The protein expression level of AIF1 after transfected with sh-AIF1 or not was detected by western blot assays. **E**-**F** Growth curves were analyzed by cell proliferation assay. **G**-**I** Wound-healing assay showed the motility ability. **J**-**K** The invasive ability of stable overexpression of RILP in osteosarcoma cells was detected by transwell assay. **L**-**M** Immunohistochemical analysis of AIF1 in normal and tumors. Scale bar: 50 μm. **N**-**P** Western blot assay was performed to detect the protein expression level of NF-κB, p-NF-κB, BCL-2, and BAX in osteosarcoma cells stably overexpressing AIF1. (**P < 0.01, ***P < 0.001, ****P < 0.0001)

To testify to what we have analyzed from datasets, we performed several experiments in osteosarcoma cells. We first detected the expression of the protein of AIF1 in 143B, MG63, HOS, and hFOB1.19 (osteoblasts cells). The results showed that the expression of AIF1 in these osteosarcoma cells was significantly decreased (Fig. 10A, B). Then we chose 143B and U2OS for further research. To study the effect of AIF1 on the proliferation of osteosarcoma cells, we increased the expression of AIF1 in 143B and U2OS cells. The transfection efficiency was evaluated by western blot (Fig. 10C, D). The 143B and U20S cells were then divided into LV-Control and LV-AIF1 groups. Cell proliferation assay revealed that LV-AIF1 strongly inhibited the growth of 143B and U2OS cells (Fig. 10E, F). We further performed a set of cell function experiments to investigate whether AIF1 overexpression can inhibit the migration and invasion of osteosarcoma. Wound-healing assay showed that the motility of LV-AIF1 143B and U2OS cells was dramatically reduced compared with LV-Control (Fig. 10G, H). In the transwell assay, AIF1 overexpression significantly decreased the invasive ability of osteosarcoma cells (Fig. 10J, K). Moreover, IHC results suggested a higher AIF1 level in the normal tissue than that in the osteosarcoma tissue (Fig. 10L, M). We further identified the effect of AIF1 on the NF-κB pathway by detecting the expression levels of NF-κB, p-NF-κB, BCL-2, and BAX using western blotting (Fig. 10N-P), and found that the expression levels of p-NF-κB and BCL-2 were significantly decreased, while that of BAX was increased. Thus, these results show that AIF1 negatively regulates the NF-κB pathway in osteosarcoma cells. Taken together, we can conclude that the NF-κB pathway plays an important role in AIF1-mediated proliferation, migration, and invasion of osteosarcoma cells. Taken together, our results suggested that overexpression of AIF1 restrained the proliferation, migration, and invasion of osteosarcoma cells.

## Discussion

Osteosarcoma is the most common primary malignant bone tumor, with an initial peak in the late adolescent and young adult period [27]. Despite significant advancements in the diagnosis and treatment of osteosarcoma to date, overall survival has remained relatively constant for the last 4 decades, especially for those patients who are diagnosed with metastatic disease and have a poorer prognosis [28, 29]. Several genetic risk factors for the development of osteosarcoma are well established and such dysregulation may represent a potent sign of new targets [21, 30, 31]. However, the molecular mechanisms of osteosarcoma remain poorly understood. Microarray technology enables us to explore the genetic alterations in osteosarcoma and has been proven to be a useful approach to identifying new biomarkers in other diseases.

Bioinformatics is an important frontier subject for the storage, retrieval, and analysis of biological information, usually genetic components (DNA and protein sequences) [32]. During the last decades, microarray technology and bioinformatics analysis have been widely used to screen genetic alterations at the genome level, which have helped us identify the differentially expressed genes (DEGs) and functional pathways involved in the carcinogenesis and progression of osteosarcoma [33–35]. Gene profile, as one of the bioinformatical technologies used for several decades, can quickly select suitable DEGs from The Cancer Genome Atlas (TCGA) and Gene Expression Omnibus (GEO), international public databases where most of the data have been deposited and stored [36].

In the present study, 3 mRNA microarray datasets and the TCGA dataset were analyzed to obtain DEGs between osteosarcoma tissues and normal osteoblasts tissues. A total of 126 DEGs were identified among the 3 datasets, including 65 upregulated genes and 61 downregulated genes. GO and KEGG enrichment analyses were performed to explore interactions among DEGs. GO enrichment analysis revealed that the DEGs mainly enriched in the platelet degranulation and extracellular exosome; while KEGG pathway analysis showed that DEGs were mainly enriched in Osteoclast differentiation, complete, and coagulation cascades. Previous studies have reported that activated platelets (activated by thyroid hormone as L-thyroxine, then promotes platelet aggregation and degranulation) may contribute to the metastatic behavior of tumor cells [37]. In addition, recent studies have revealed that exosomes have activities as diverse as remodeling the extracellular matrix and may play important roles in human cancers [38]. Moreover, several researchers have brought forward a tumor-promoting role for osteoclast differentiation [39–41]. In a word, all these theories are consistent with our results. Likewise, module analysis showed that genes in this module were mainly enriched in osteoclast differentiation, integrin-mediated signaling pathway, and *Staphylococcus aureus* infection.

We selected 4 DEGs as hub genes with degrees ≥10 and assessed the expression of these genes about overall and metastasis-free survival using the online GSE21257 dataset. Among these hub genes, only AIF1 showed a statistical difference between patients who developed metastasis within 5 years and those who didn’t. To be specifically elucidated, osteosarcoma patients with high expression of AIF1 alteration showed better overall survival and metastasis-free survival. This finding led us to guess whether AIF1 could be a regulator of osteosarcoma. In the previous literature, we know that AIF1 plays a role as an actin-binding protein that enhances membrane ruffling and RAC activation. In addition, it plays a role in RAC signaling and phagocytosis. AIF1 also has such functions: 1) may play a role in macrophage activation and function; 2) promotes the proliferation of vascular smooth muscle cells and of T-lymphocytes; 3) enhances lymphocyte migration; 4) plays a role in vascular inflammation [1, 5, 8]. Surprisingly, scientists have discovered the role of AIF1 in regulating cancer in recent years. Yang provided evidence that the level of AIF-1 expression may serve as a protective prognostic indicator for gastric cancer by regulating β-catenin [10]. Ferial suggested that AIF1v1 as much as AIF1v3 plays a major role in the crosstalk between breast cancer and infiltrating immune cells mediating tumor progression, implying their high potential as target molecules for breast cancer diagnosis, prognostication, and treatment [7]. Zhang revealed that AIF1 was upregulated in hepatocellular carcinoma. Silencing of AIF1 expression resulted in a reduction in cell proliferation and migration in human hepatocellular carcinoma cells [11]. In addition, Ai-founded AIF1 plays the role of a tumor suppressor in colorectal cancer cells by inhibiting cell proliferation, suppressing cell migration, and inducing cell apoptosis. AIF-1 overexpression promoted the apoptosis of colorectal cancer cells by activating the p38 MAPK pathway [12]. Combining these studies and our results, we found that AIF1 may be an osteosarcoma guardian gene and may serve as a protective prognostic indicator.

Therefore, we investigated the AIF1 gene again. According to the expression level of AIF1, osteosarcoma patients in TCGA were divided into high- and low-expression groups, and then differential genes between the two groups could be obtained. GO and KEGG enrichment analysis of differential genes showed that AIF1 was closely related to the immune and NF-kappa B signaling pathways of osteosarcoma. To verify the relationship between AIF1 and immunity, we conducted GSEA and ssGSEA analyses and found that AIF1 acts on many immune-related pathways, such as antigen processing and presentation, natural killer cell mediated cytotoxicity, complement and coagulation cascades, and B cell receptor signaling pathway. AIF1 is also related to tumor microenvironment score, such as the high-expression AIF1 exhibited significantly higher immunological scores, estimate scores, stromal scores, and lower tumor purity. Analysis of immune infiltration in patients with osteosarcoma showed that AIF1 can affect the expression of many immune cells and immune functions. Finally, we analyzed the relationship between AIF1 and known immune checkpoints and found that AIF1 is associated with some immune checkpoints such as HVEM, TIM-3, TIGIT, CD200R1, and CTLA4. Finally, we carried out a series of cell experiments. First, we found that AIF1 was lowly expressed in osteosarcoma cell lines, especially in 143B and U2OS cells. So we chose 143B and U2OS cells for the following experiments. We first constructed an AIF1-overexpressing cell line in 143B and U2OS cells with a recombinant lentivirus. We then found that the proliferation rate, invasion rate, and mobility of LV-AIF1 osteosarcoma cells were significantly inhibited, while apoptotic protein expression was increased and apoptosis was increased. These results are similar to the effects produced by knocking down AIF1 expression in gastric cancer [10]. We subsequently found that AIF1 can inhibit the expression of the NF-kappa B pathway and affect the expression of proteins in the NF-kappa B signaling pathway. In summary, we found that AIF1 is related to the immunity of osteosarcoma, affects the immune-related pathway, and inhibits the occurrence and development of osteosarcoma by inhibiting the NF-kappa B signaling pathway.

This is the first study to report the association between AIF1 expression and the clinical pathological features of osteosarcoma. Our data demonstrated that AIF1 functions as a tumor suppressor, and loss of AIF1 expression may represent a novel indicator for the progression and prognosis of osteosarcoma. However, the specific mechanisms by which AIF1 inhibits tumor action by inhibiting the NF-kappa B signaling pathway, and whether AIF1 is beneficial as a future prevention and treatment target for osteosarcoma, remain to be further investigated.

### Supplementary Information

**Supplementary Material 1.**

## Data Availability

We obtained the datasets analyzed and generated during the current study in the TCGA GDC repository (https://portal.gdc.cancer.gov) and GEO repository (https://www.ncbi.nlm.nih.gov/geo/). All data included in this study are available upon request by contact with the corresponding author.
