# Supplementary material for: Identification of allograft inflammatory factor-1 suppressing the progression and indicating good prognosis of osteosarcoma
Source: BMC Musculoskelet Disord. 2024 Mar 23;25:233. doi: 10.1186/s12891-024-07363-8 (PMC10960474; doi:10.1186/s12891-024-07363-8)
Supplement: Supplementary file 1 — Supplementary Material 1. [file 12891_2024_7363_MOESM1_ESM.pdf]

Identification of allograft inflammatory factor-1 suppressing the progression and indicating good prognosis of osteosarcoma

Supplementary Figure

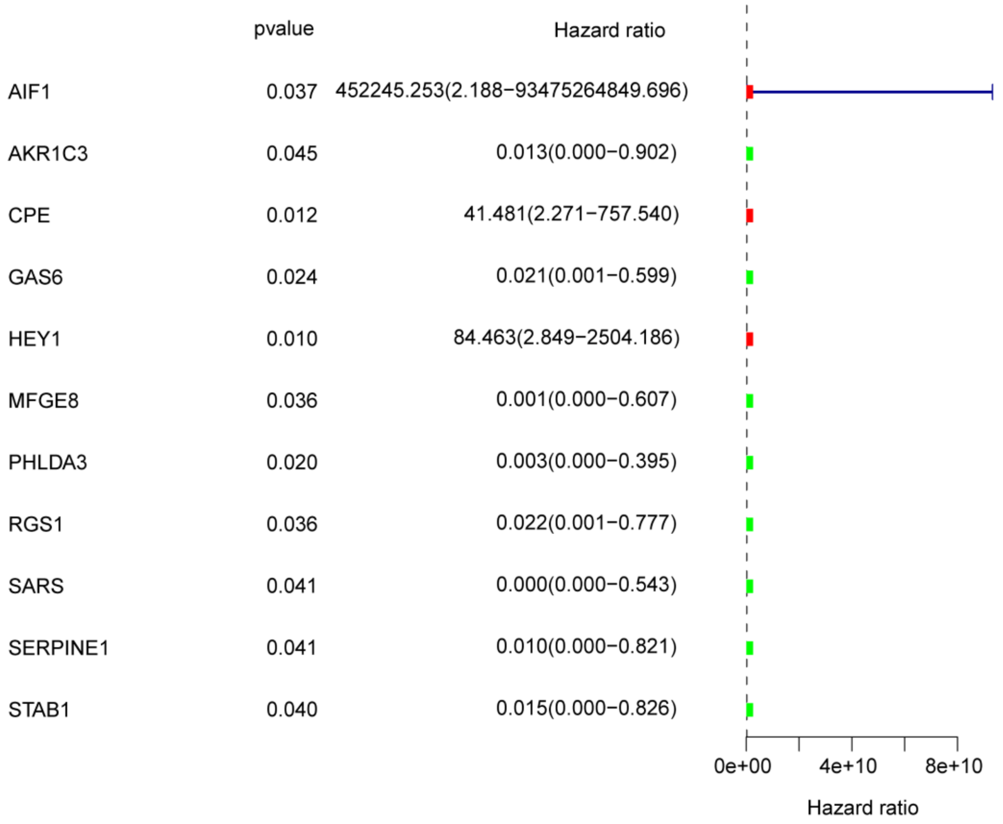

Supplementary Figure S1. Cox analysis of the independent risk factor of osteosarcoma.

Original blots

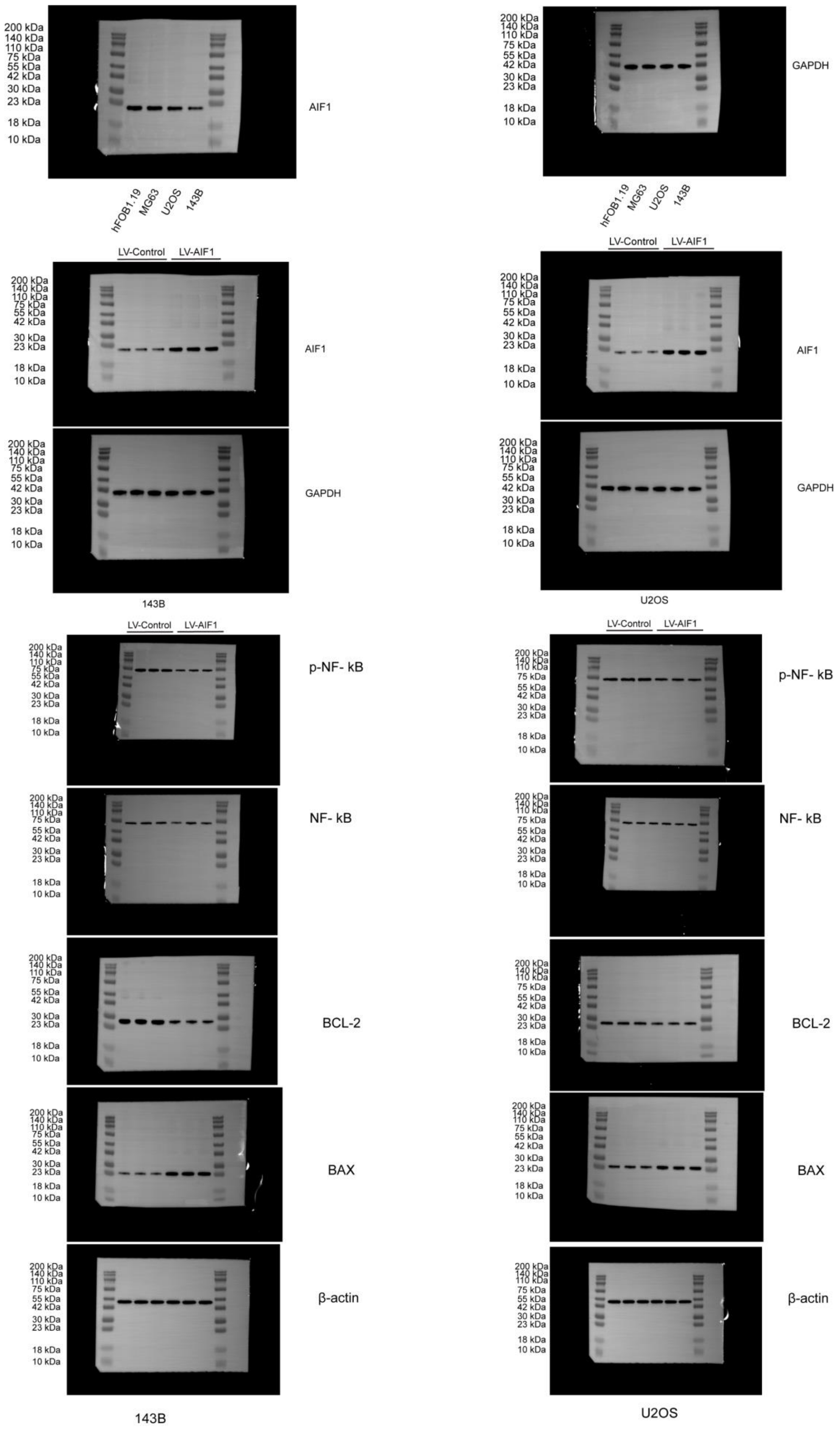
200 kDa
140 kDa
110 kDa
75 kDa
55 kDa
42 kDa
30 kDa
23 kDa
18 kDa
10 kDa
AIF1
GAPDH
hFOB1.19
MG63
U2OS
143B
LV-Control
LV-AIF1
AIF1
GAPDH
143B
U2OS
p-NF- kB
NF- kB
BCL-2
BAX
β-actin
143B
U2OS
